# A Sensor Image Dehazing Algorithm Based on Feature Learning

**DOI:** 10.3390/s18082606

**Published:** 2018-08-09

**Authors:** Kun Liu, Linyuan He, Shiping Ma, Shan Gao, Duyan Bi

**Affiliations:** College of Aeronautics Engineering, Air Force Engineering University, Xi’an 710038, China; mashiping@126.com (S.M.); 17709223919@163.com (S.G.); biduyan@126.com (D.B.)

**Keywords:** image dehazing, feature learning, sparse coding, generative adversarial networks

## Abstract

To solve the problems of color distortion and structure blurring in images acquired by sensors during bad weather, an image dehazing algorithm based on feature learning is put forward to improve the quality of sensor images. First, we extracted the multiscale structure features of the haze images by sparse coding and the various haze-related color features simultaneously. Then, the generative adversarial network (GAN) was used for sample training to explore the mapping relationship between different features and the scene transmission. Finally, the final haze-free image was obtained according to the degradation model. Experimental results show that the method has obvious advantages in its detail recovery and color retention. In addition, it effectively improves the quality of sensor images.

## 1. Introduction

Visual information accounts for more than 80% of the total amount of information that is available to humans. As an extension of the visual system, image sensors [1] play an increasingly important role in industry, agriculture and daily life. Different sensors are widely used in military, astronomy, medical, radio, television, fax and communication applications due to their high integration, low power consumption, impact resistance and long life. However, in bad weather conditions such as foggy weather, external scene information is seriously polluted, the color of the perceived scene is seriously drifted, the contrast and saturation performance are rapidly degraded, and the features of the target are difficult to recognize. Therefore, how to use digital processing technology to avoid the above problems and improve the sensor’s ability to perceive the real world has become an urgent problem that must be solved.

Image dehazing aims to recover real scene information covered by fog in sensor images through relevant technical means and methods, to obtain complete details, structural information and natural, haze-free images. To improve the quality of images obtained by the sensors, many dehazing methods have been proposed.

Currently, image dehazing methods are mainly divided into the methods based on image enhancement and the methods based on image restoration. The former include: histogram equalization [2], homomorphic filtering [3], wavelet transform [4] and Retinex methods based on color invariance [5]. The image enhancement-based dehazing method improves the contrast of the restored image and highlights local features such as the details of the haze image. However, it does not consider the fundamental cause of the degradation of the haze image. therefore, the restored image cannot effectively reflect the real information of the scene, leading to the drawbacks of incomplete dehazing and severe color distortion.

On the other hand, methods based on image restoration have been widely used in recent years: He [6] proposed the dark channel to estimate transmission, and this method has been widely adopted. However, color distortion occurs in restored results for large white areas such as the sky. According to the degeneration model and atmospheric scattering model, Tan [7] proposed a dehazing method for maximizing local contrast based on the assumption that local area atmospheric light is smooth. However, the restored images show halo effects and supersaturation.

Many other improved methods have been proposed: Zhu [8] proposed the use of color attenuation and estimated the transmission through the linear model, which improved the operation efficiency of the method. However, his method relied too much on image color information, resulting in an unsatisfactory effect when testing the entire haze image. Berman [9] estimated transmission by calculating the color distance point-by-point. This method effectively avoided block effects, but it is prone to color saturation. Tang [10] adopted random forest to combine dark primary colors with multiple color features to improve the accuracy of transmission. However, this method did not account for the textural features associated with haze, resulting in inaccurate estimation of the transmission.

It is apparent from the above method that most current methods are only based on color features or structure features. To better utilize features such as texture, structure, and color for improving the dehazing effect, we propose an image dehazing algorithm based on feature learning. Using sparse coding and GAN as tools, our method aims to explore the mapping relationship between transmission and color and structure features of the images to achieve accurate estimation of transmission. The main contributions of the paper are as follows:

Unlike most other methods, we consider the effects of color and structure features while estimating the transmission map. Through the training process of a weighted sparse dictionary, the multiscale texture and structure features of the images are extracted. At the same time, the multiscale color features of the image are considered in the estimation of the transmission.

We investigated the mapping relationship between features and the transmission through generative adversarial networks. In addition, the model of our networks was jointly constructed with a multilayer neural network and a convolutional neural network to further ensure the accuracy of estimation.

The remaining sections are arranged as follows: in Section 2, we introduce the generative process of haze images and apply it to the generation of training samples. In Section 3, we explain how to extract the color and structure features. Moreover, we build the GAN model to obtain the mapping relationship between the transmission and the features in this section. In Section 4, we further prove the effectiveness of our method in detail recovery and color preservation through experiments. In Section 5, we summarize the paper and provide future focus.

## 2. The Degradation Model and the Test Database

In this section, we introduce the foggy degradation model and the process of building a test database based on the degradation model.

### 2.1. Degradation Model

**Figure 1:**
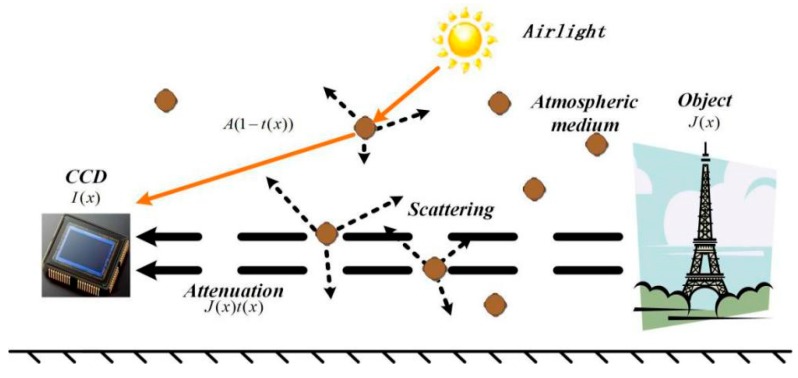
Degradation model.

The degradation model was proposed by McCartney [11] in 1978, and has been widely used in computer vision and image processing. The model describes the objective mechanism of haze image degradation. The scattering effects that cause image degradation are divided into two categories: One is the direct attenuation, wherein reflected light on the surface of the target is attenuated by suspended particles in a turbid atmospheric medium. The other is the indirect attenuation, wherein atmospheric light is scattered through the suspended particles. The degradation model of the images acquired by the CCD sensors is shown in Figure 1.

In Figure 1, *I*(*x*) represents haze image acquired by the CCD sensor, *J*(*x*) represents the reflected image of the scene, which denotes the haze-free image to be restored, and A denotes the global atmospheric light intensity. In our paper, we assume that the atmospheric light value is constant. Furthermore, *t*(*x*) represents the transmission, reflecting the attenuation degree of the scene, which can be expressed as:

(1) $$t(x)=e^{-\beta d(x)}$$

where *β* denotes the atmospheric scattering rate and *d*(*x*) denotes the depth of scene, indicating the distance from the target to the CCD sensors. He [6] established a foggy image degradation model as follows:

(2) I(x)=J(x)t(x)+A(1−t(x))

where *J*(*x*)*t*(*x*) is the interference term due to direct attenuation, and *A*(1 − *t*(*x*)) is the interference term due to indirect attenuation.

The accurate estimation of transmission *t*(*x*) is the key to obtain the haze-free image *J*(*x*). The method in our paper focuses on obtaining accurate transmission through feature extraction and generative adversarial networks. Since we assume that A is a constant, which is consistent with the literature [6], we can obtain the haze-free image *J*(*x*) according to Equation (3) when *t*(*x*) is obtained:

(3) $$J(x)=\frac{I(x)-A}{\max(0.1,t(x))}+A$$

To reduce the introduction of noise, we set 0.1 as the minimum value of the transmission, which is consistent with the parameter setting in He [6] and is widely used by most methods now.

### 2.2. Training Sample Generation of Our Networks

Since there is no publicly available dataset containing haze-free images and its corresponding haze images and transmission for the training of our networks, we selected an image from the NYU databases as the dataset. The NYU dataset [12] consists of video sequences from a variety of indoor scenes, recorded by RGB and depth cameras from Microsoft Kinect, which have high accuracy. It is a standard training dataset with haze-free images of different scenes and real depth maps. It has been used in research regarding image segmentation widely, detection and recognition. The NYU dataset is appropriate for use in network training in this study.

**Figure 2:**
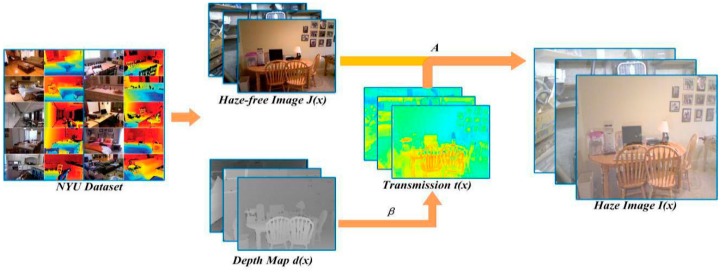
The formation of haze images.

According to the degradation model, we randomly selected 500 haze-free images and their true depth maps from the NYU dataset to generate the corresponding haze images and transmission to be used as training samples. The formation of haze images is shown in Figure 2.

First, we selected the true depth map and the haze-free image from the NYU database. Meanwhile, an atmospheric scattering coefficient *β* is generated randomly, which ranges from 0.5 to 1.5. The selection of *β* is based on the experience of the most advanced algorithms, which can help us get accurate transmission. What’s more, the large range of *β* also enriches the sample database, we can obtain haze images with different density. According to Equation (1), we can obtain the transmission *t*(*x*), which has been regarded as ground truth *T* in our paper. And then, the haze image *I*(*x*) can be obtained according to Equation (2).

According to this method, we obtain four groups of training samples: the haze-free image data set *J^set^* = {*J*_1_, *J*_2_, …, *J_n_*} and its scene depth map dataset *d^set^* = {*d*_1_, *d*_2_, …, *d_n_*}, and the generated haze image dataset *I^set^* = {*I*_1_, *I*_2_, …, *I_n_*} and the ground truth dataset *T^set^* = {*T*_1_, *T*_2_, …, *T_n_*}. Therefore, *J^set^*, *T^set^*, *I^set^* and *d^set^* are the training samples for the networks.

## 3. Framework on Transmission Restoration

**Figure 3:**
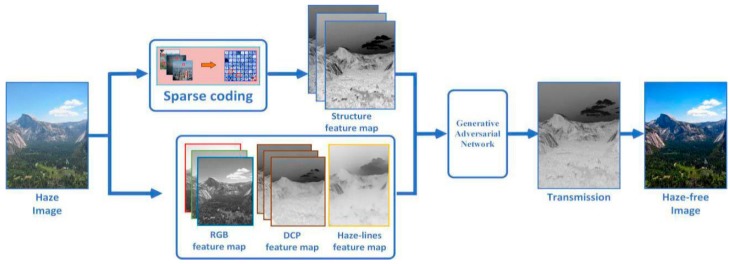
The flow of our method.

In Section 3, we explain the framework of our algorithm in detail. Based on sparse coding and feature learning, we present an efficient method for estimating the scene transmission of a haze image. Figure 3 shows the flow of our method, which consists of three parts: estimating the color feature maps of haze images through different priors and assumptions, extracting the structure features of haze images through sparse coding, and estimating the scene transmission through generative adversarial networks based on the extracted color and structure features.

### 3.1. Multiscale Color Feature Extraction

As is widely known, there are great differences in color between haze images and haze-free images. Therefore, color features are often used to estimate the scene transmission of a haze image in image dehazing. A variety of color-based priors are fully adopted in a single image removal, such as the color-lines prior proposed by Fattal [13], the dark channel prior (DCP) proposed by He [6], and haze-lines prior proposed by Berman [9]. The different kinds of priors can be viewed as a series of color features related to haze density. In fact, although these features can achieve image dehazing effectively, different features have unique limitations. For example, images restored through DCP show color distortion in the sky region. To improve the accuracy of the scene transmission, a variety of color features have been extracted from the haze images to further estimate the transmission in our method: multiscale dark primary color features, haze-lines color features, and RGB channel color features.

#### 3.1.1. Multiscale Dark Primary Color Features

**Figure 4:**
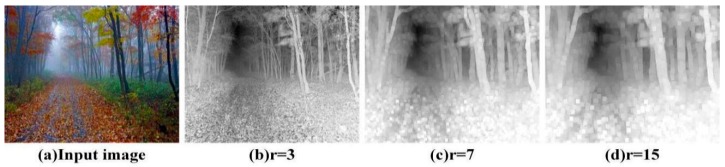
The DCP color feature maps.

In image dehazing, dark primary color is an important color feature. In most non-sky local areas, some pixels always have very low values in at least one color channel. In this paper, the dark primary color features are defined as:

(4) $$P_{r}(x)=1-\frac{\underset{y\in \Omega (x)}{\min}(\underset{C\in \{R,G,B\}}{\min}I^{c}(x))}{A}$$

where Ω(*x*) denotes the local patches centered on *x*, of which the size is *r* × *r*. *I^c^*(*x*) represents the components of haze image *I*(*x*) under the color channel *C*. *P_r_*(*x*) denotes the DCP color feature map, which denotes the dark primary color features of local patches of the size of *r* × *r* and is used to estimate the scene transmission *t*(*x*). Since *r* is directly related to the estimation of the transmission *t*(*x*), we adopt multiscale dark primary color features to estimate the transmission *t*(*x*). Different scales of dark primary color features can reflect more information of the scene transmission *t*(*x*). Different scales of the DCP color feature maps *P_r_*(*x*) are shown in Figure 4. It is apparent that the DCP color feature maps approximately reflect the distribution of haze in the image. The brighter the area in the image, the higher the haze density is. At the same time, *r* has an important influence on the shape of DCP color feature maps.

#### 3.1.2. Haze-Lines Color Features

Berman [9] clusters the RGB values of individual images in the Berkeley Segmentation Database, which contains a variety of haze-free images. It is found that a clear natural image can be represented by hundreds of different RGB values at most, and different clusters can be formed in RGB space. For a given cluster, the pixels belonging to the cluster are nonlocal and distributed in different locations of the entire image. Under the influence of haze, the distance from pixels located in different regions of the image to the camera is different. The pixels that belong to the same color cluster finally correspond to different RGB values instead of clustering into one cluster. There is a line in RGB space called the haze-line. We can estimate the transmission according to the color features in the image and the position of the pixels on its haze-line.

We define the haze-line feature map *C*(*x*) and *I_A_*(*x*) based on the non-local distribution of pixels in RGB space and mapping it into a spherical coordinate system:

(5) $$\begin{array}{l} I_{A}(x)=C(x)\cdot [J(x)-A]\\ I_{A}(x)=[r(x),\theta (x),\varphi (x)] \end{array}$$

where *r*(*x*) represents the distance from the pixel to atmospheric light A. Berman assumed that *θ*(*x*) and *ϕ*(*x*) are constant in the same haze-line. The transmission is only related to the distance of color. Based on the above assumption, we can summarize using the following expression:

(6) $$C(x)=\frac{C(x)\left\|J(x)-A\right\|}{\left\|J(x)-A\right\|}=\frac{r(x)}{r_{\max}}$$

where $r_{\max}=\underset{x\in L}{\max}\left\{r(x)\right\}$, *L* represents haze-lines, and *C*(*x*) represents the relationship between the color features of the image and the transmission *t*(*x*).

#### 3.1.3. RGB Channel Color Features

Since most of the color features are based on the RGB color model, the three components (R channel component, G channel component and B channel component) of the haze image in RGB space are of great value to the estimation of transmission *t*(*x*) [14]. We define the RGB feature maps *K_c_*(*x*) as:

(7) $$\begin{array}{l} K_{r}(x)=I_{red}\\ K_{g}(x)=I_{green}\\ K_{b}(x)=I_{blue} \end{array}$$

where *K_c_(x)* represents three components of the haze image in RGB color space. As special feature maps, RGB feature maps have been important reference for the estimation of transmission *t*(*x*).

### 3.2. Multiscale Structure Feature Extraction

Since the traditional dehazing algorithm simply depends on the color feature to estimate transmission *t*(*x*), it has some limitations. The texture and structural features of the object are important information for estimating the transmission *t*(*x*), especially for objects with regular shapes or light colors. As a result, the texture and structure of haze images play a key role in the estimation. In this paper, sparse coding is used to extract texture and structural features of haze images, to further estimate the transmission *t*(*x*). Sparse coding [15] is an unsupervised learning method, which is used to find a set of super complete base vectors to represent sample data more efficiently. It can realize the automatic selection of features and remove the features without information through learning. In other words, it resets the corresponding weights of these features to 0. Because it can extract the main features of objects effectively, it is widely used in object detection and recognition.

**Figure 5:**
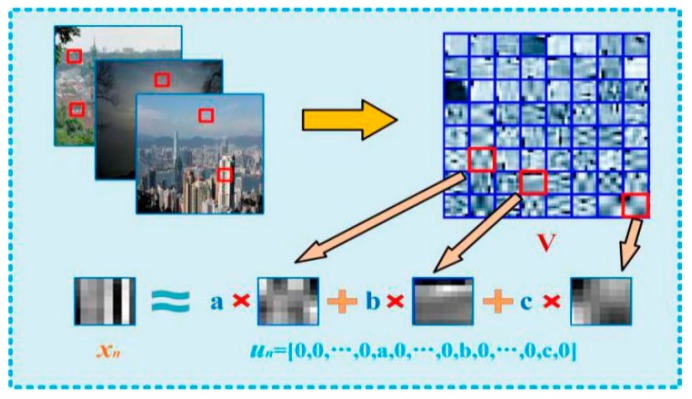
Dictionary training.

The sparse coding of natural images and the process of dictionary training are shown in Figure 5. *V* represents the visual dictionary, *x_n_* represents a local patch of the image, and *u_n_* represents the sparse coefficients that correspond to *x_n_*. It is apparent that *x_n_* is represented as linear combinations of different atoms of dictionaries *V*. The process of sparse coding is expressed as follows:

(8) $$\underset{U,V}{\min}\sum_{n=1}^{N}{\left\|x_{n}-u_{n}V\right\|}_{}^{2}+\lambda {\left\|u_{n}\right\|}_{1}^{}$$

where $\lambda {\left\|u_{n}\right\|}_{1}^{}$ denotes a regularization constraint for *u_n_*, *λ* denotes a regularization parameter and ${\left\|u_{n}\right\|}_{1}^{}$ denotes a coefficient penalty term. The sparser the coefficient vector, the smaller the value of the target equation. As such, in the optimization process, the coefficient vector will evolve in a more sparse direction. The essence of sparse coding is that a target vector *x_i_* can be linearly fitted by a small number of base vectors. In this paper, the feature map of the image is divided into three steps.

• Dictionary Training

In this process, we test a large number of training samples to obtain a set of redundant basis vectors through unsupervised learning, which is also called redundant dictionary *V*. The dictionary V reflects some essential elements in the training sample, such as the edges and corners of the image. Experiments show that the dictionary learning process simulates the processing of information in the human visual cortex. The calculation of dictionary *V* can be expressed as the least square problem with quadratic constraints, that is:

(9) $$U,V=\underset{U,V}{\arg\min}{\left\|X-UV\right\|}_{2}^{2}+\lambda {\left\|U\right\|}_{1}$$

• Linear fitting

An arbitrarily local patch *x_n_* which converts to the form of vector can be fitted by a linear combination of several entries in the dictionary *V*. According to different constraint conditions, we obtain a different fitting coefficient *u_n_*, such that the image features can be expressed with the coefficient vector *u_n_*. Since *V* is known, we perform sparse coding on image *X* to obtain the corresponding coefficient matrix *U*, the upper type is rewritten to:

(10) $$\underset{u_{n}}{\min}{\left\|x_{n}-u_{n}V\right\|}_{}^{2}+\lambda {\left\|u_{n}\right\|}_{1}^{}$$

We adopt a K-SVD dictionary learning algorithm to train the dictionary *V* and the sparse coefficient matrix *U*, and the objective functions are as follows:

(11) $${\left\|X-UV\right\|}_{2}^{2}={\left\|X-\sum_{j=1}^{k}v_{j}u_{j}\right\|}_{2}^{2}={\left\|(X-\sum_{j\neq k}^{}v_{j}u_{j})-v_{k}u_{k}\right\|}_{2}^{2}={\left\|E_{k}-v_{k}u_{k}\right\|}_{2}^{2}$$

where *v_j_* denotes the vectorized atom that belongs to dictionary *V*, *u_j_* denotes the coefficient vector of the local patch, and *E_k_* is an error matrix. Then, we update the dictionary column by column and the corresponding nonzero sparse coefficient is updated, by which the dictionary *V* and the sparse coefficient matrix *U* are obtained.

• Feature weighting

**Figure 6:**
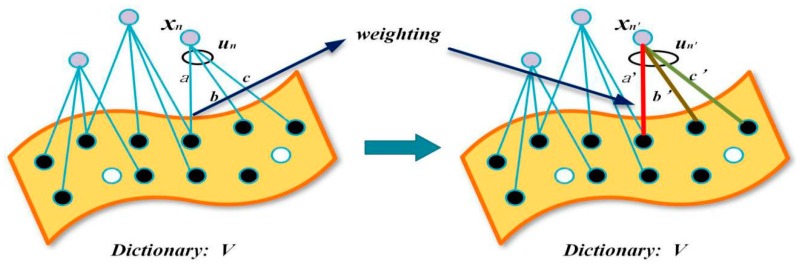
Dictionary training.

We use weighted sparse coding to extract texture and structure features of images. By weighting the sparse coefficient matrix *U*, the texture and detail of the images are further highlighted, which increases the weight of texture and structural features and makes texture and structure more prominent in the estimated feature map. As such, the reconstructed feature map *S_l_*(*x*) is obtained, where *l* represents the size of local image patch that has been trained. Additionally, the relationship between texture features and the scene transmission graph is more intuitively represented. The weighted sparse coefficients are as follows:

(12) $${u_{n}}^{\prime }=\frac{1}{Z}\frac{\left|u_{n}\right|}{\sum_{i=1}^{k}{\left|u_{i}\right|}_{}}u_{n}$$

(13) $$Z=\max\frac{\left|u_{n}\right|}{\sum_{i=1}^{k}{\left|u_{i}\right|}_{}}u_{n}$$

where *Z* denotes the normalized coefficient, which makes *u_n_*′ range from 0 to 1. And *u_n_*′ denotes a weighted sparse coefficient matrix. The diagram of coefficient weighting is shown in Figure 6:

where *x_n_* denotes the local image patch, *u_n_* = (0, 0, …, 0, a, 0, …, 0, b, 0, …, 0, c, 0) and *u_n_*′ = (0, 0, …, 0, a′, 0, …, 0, b′, 0, …, 0, c′, 0). *x_n_*′ denotes the weighted local image patch*.* Once the dictionary *V* and the coefficient matrix *U’* are known, we can obtain the weighted structure feature map *S_l_*(*x*) by sparse coding. To estimate the transmission accurately, we train the image blocks of different sizes through the dictionary, and construct the multiscale structure feature map *S_l_*(*x*) (*l* = 3, 8, 13), which is the same as DCP color feature maps *P_r_*(*x*).

**Figure 7:**
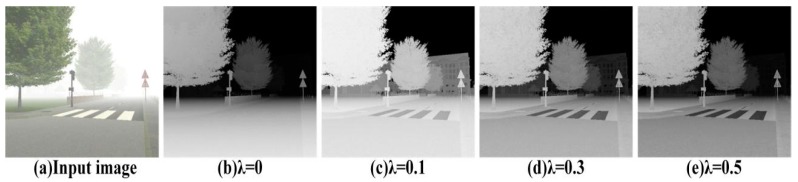
the structure feature maps with different *λ.*

In addition, to analyze the effect of the value of *λ* on the structure feature maps, experiments were carried out with *λ =* 0, 0.1, 0.3 and 0.5 respectively. As we can see in the Figure 7. The structure feature map with *λ =* 0.1 has no detail loss and has appropriate brightness. So we selects *λ =* 0.1 to estimate the structure feature maps.

### 3.3. Estimating the Scene Transmission through GAN

According to the extracted color and structure features, we construct a mapping network between different types of features and the transmission *t*(*x*), which can achieve effective estimation of scene transmission. Based on the GAN, we obtain the transmission *t*(*x*) by mapping the input features to the multilayer network. The network framework is divided into two parts: generative networks *G*(*) and adversarial networks *D*(*). Through the generative networks *G*(*), we construct a mapping network between the color and structure features of haze images and transmission *t*(*x*). We thus obtain the generative transmission *t_g_*(*x*). Meanwhile, the adversarial networks D(*) discriminate and classify the input generative transmission *t_g_*(*x*), to form a whole Generative adversarial network.

#### 3.3.1. Generative Networks

Since the color and structure features are very important to the estimation of the transmission *t*(*x*), we assume that there is a nonlinear mapping relationship between the various features and the transmission *t*(*x*), which can be expressed as:

(14) $$t_{g}(x)=f_{1}(f_{2}(\cdot \cdot \cdot f_{n}(F_{1}(x),F_{2}(x),\cdot \cdot \cdot ,F_{k}(x))))$$

where *x* represents a particular position in the image and *F_k_* denotes the *k^th^* feature map. In our generative networks, *k* = 10, and *F*_1_, *F*_2_, …, *F*_10_ represent the *S*_3_(*x*), *S*_8_(*x*), *S*_13_(*x*), *K_r_(x)*, *kg(x)*, *K_b_(x)*, *P*_3_(*x*), *P*_7_(*x*), *P*_15_(*x*), *C*(*x*) defined in Section 3.1 and Section 3.2. *f*_1_(*), *f*_2_(*), …, *f_n_*(*) denotes a series of unknown nonlinear functions. *t_g_*(*x*) represents the generative transmission *t_g_*(*x*). Then, we establish a multilayer generative networks model. Next, the sample sets *I^set^* and *T^se^***^t^** are trained for further determination of *f*_1_(*), *f*_2_(*), …, *f_n_*(*). Finally, we estimate the *t_g_*(*x*) of input haze images by using a trained multilayer generative network.

**Figure 8:**
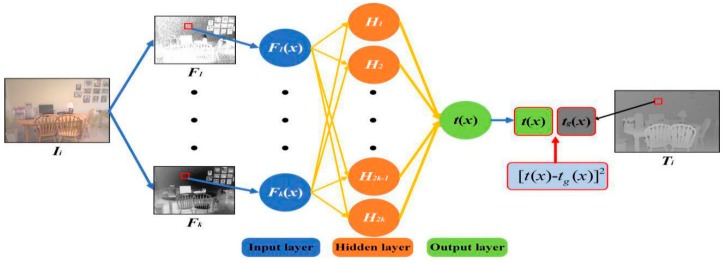
The generative network.

The generative network model and its training process for the estimation of scene transmission is shown in Figure 8. The network consists of an input layer, a hidden layer and an output layer. The number of neurons in the input layer is equal to the number of feature maps. There are twice as many neurons in the hidden layer as in the input layer, and there is only one neuron in the output layer, which represents the generative transmission *t_g_*(*x*).

The training process of the network is as follows: we extract different features of haze images *I*_1_, *I*_2_, *…*, *I_n_*, from the training sample set *I^set^.* Each haze image *I_i_* corresponds to *k* feature maps. Then, we choose these feature maps as input images, and ground truth *T_i_* in the *T^set^* as labels. Finally, the back propagation (BP) algorithm [16] is used for supervised learning of the networks. Thus, we obtain the trained generative network model. Through the trained network model, we can estimate the generative transmission while inputting a haze image:

(15) $$H_{j}=f(\sum_{i=1}^{k}F_{i}(x)W_{2_{i,j}}^{\left(1\right)})$$

(16) $$H_{j}=f(\sum_{i=1}^{k}F_{i}(x)W_{2_{i,j}}^{\left(1\right)})$$

(17) $$f(z)=\frac{1}{1+\exp(-z)}$$

(18) $$G(I_{i})=f(\sum_{j=1}^{2k}f(\sum_{i=1}^{k}F_{i}(x)W_{2_{i,j}}^{\left(1\right)})W_{2_{j,1}}^{\left(2\right)})$$

where $W_{2_{p,q}}^{\left(l\right)}$ represents the weights from the *p*th neurons in the *l* layer network to the *q*th neurons in the next layer network, *f*(*) means sigmoid functions. As a kind of activation function. It is not only simple to calculate, but also can effectively suppress the error. *H_j_* donates the *j*th output in the hidden layer, and *t_g_*(*x*) and *G*(*I_i_*) are the output images. In other words, *G*(*I_i_*) is equal to *t_g_*(*x*).

#### 3.3.2. Adversarial Networks

**Figure 9:**
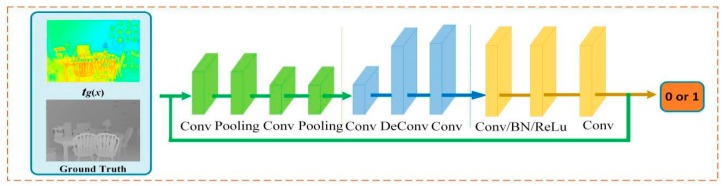
The adversarial networks.

The adversarial networks mainly classify the input transmission and obtain the correct discriminant probability. Since there is a certain correlation between the transmission and depth information of the scene, we extract the features of the transmission through a convolutional neural network, which helps us obtain the characteristics of the transmission. Then, we regard the characteristic as the criterion of discrimination of the transmission. In this way, we obtain the discriminating probability of two kinds of transmissions. The adversarial networks model is shown in Figure 9.

The BN layer and Relu activation layer are applied to all convolution layers of the adversarial networks. We perceive the input information and extract the haze features of the transmission. The output is 1 if it is a ground truth, whereas it is 0 if it is the generative transmission *t_g_*(*x*).

#### 3.3.3. Loss Function.

To form the overall confrontational training network and improve the robustness of the overall network, *G*(*) and *D*(*) should satisfy the following loss function:

(17) $$\underset{G}{\min}\;\underset{D}{\max}\;L\;(D,G)=E_{T∼P_{data}(T)}[\log D(T)]+E_{t_{g}(x)∼P_{t}(t_{g}(x))}[\log(1-D(G(I)))]$$

where *T* represents the ground truth obtained from the training sample, which has been set as the discriminant transmission in the process of discrimination. Meanwhile, *T* satisfies the *P_data_* distribution*. t_g_*(*x*) denotes the generative transmission obtained through the generative networks, which obeys the *P_t_* distribution. *I* is the input image. Adversarial networks aim to discriminate ground truth *T* and the generative transmission *t_g_*(*x*). Generative networks aim to minimize (1 − *D*(*G*(*I*)), such that the adversarial networks cannot correctly distinguish the generative transmission. As a result, *t_g_*(*x*) is closer to the real transmission *T*. Therefore, the final transmission *t*(*x*) is estimated through the GAN, and we can obtain the haze-free image according to Equation (3).

## 4. Comparison Experiments

To illustrate the effectiveness of our algorithm, we compare the transmission and restored results of different methods, which proves the superiority of our method in the estimation of the transmission. Then, we compare the experimental results with to advanced methods in detail recovery and color preservation, respectively. Finally, we select synthetic haze images as input images to prove the applicability of our method. In addition, we further illustrate the advantages of our method by analyzing the objective index of the experimental results. All of the experimental results were completed in MATLAB 2016a on an Intel Core i7 CPU equipped with 16 GB RAM.

### 4.1. Qualitative Results

**Figure 10:**
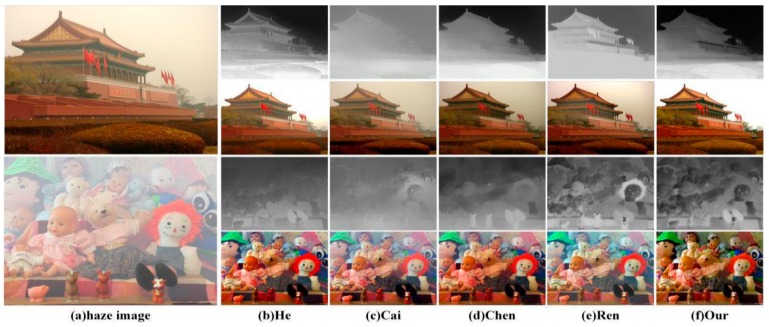
A comparison of the transmission.

The comparison of the transmission is shown in Figure 10. In the first row of Figure 10, it is apparent that the edges of the transmission estimated by using our method are clear and hierarchical. In contrast, the transmissions estimated by using the methods of Cai [17], Chen [18] and Ren [19] show detail loss. For example, the mounds in the bottom-right corners of the images disappeared. As a result, the haze-free images restored by using the three methods have a problem associated with edge blurring in the second row of Figure 10. Although the transmission estimated by using the method of He [6] is full of details, it is too light, so the bottom of the restored image shows color distortion. For the third row of Figure 10, the transmission estimated by using our method has more detail information than the other methods. The other four methods show loss of detail loss. Additionally, our experimental results are clear in detail and natural in color.

**Figure 11:**
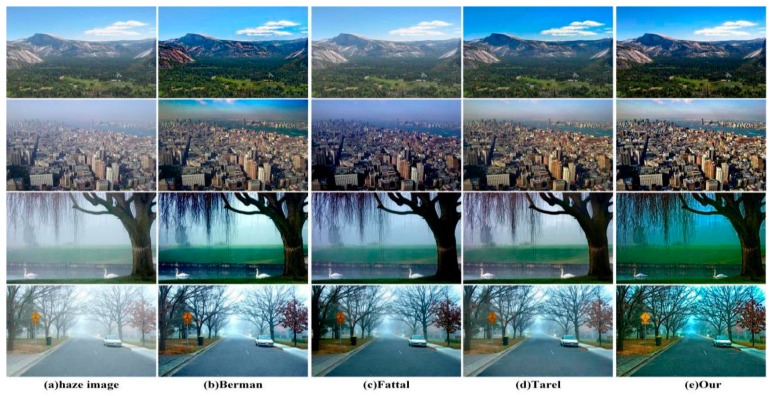
A comparison in detail recovery.

To prove the effectiveness of our method in detail recovery, we selected four challenging defogged natural images for experimental comparison. In the first row of Figure 11, the mountain restored by using the methods of Fattal [13] and Tarel [20] were covered with haze. In contrast, the details of the mountain restored by using the method of Berman [9] disappeared. In the second row of Figure 11, only our method and Berman’s method restore the clouds in the sky. However, there is color distortion in the city restored by using the method of Berman [9]. Throughout the entire image, the result restored by our method is sharper at the edges, which is pleasing to the human eye. In the third row of Figure 11, the branches restored by using our method are not only denser, but the trunk is clearly more visible than when using other methods. In addition, the restorations using the methods of Berman [9], Fattal [13] and Tarel [20] were covered with residual mist. In the final row of Figure 11, it is obvious that our method is better in haze removal for the end of the road. In conclusion, our method has obvious advantages in detail recovery and structure maintenance compared to other methods.

**Figure 12:**
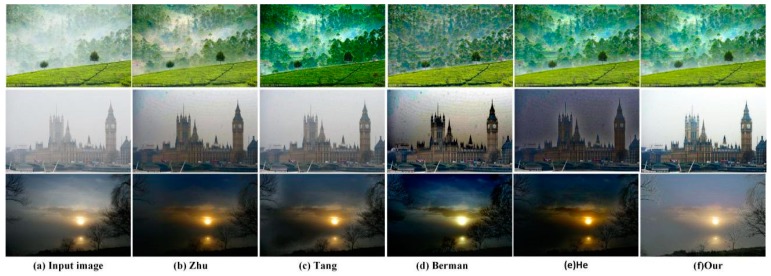
A comparison of color preservation.

Next, we compared experimental results obtained from color priors, such as those presented in He [6], Zhu [8], Tang [10], and Berman [9]. As displayed in Figure 12, we chose natural images with thick fog, a large sky area and low brightness for experimental comparison. As shown in Figure 12b, the image restored using the method of Zhu [8] is darker and is not completely dehazed. The images restored using the method of Tang [10] have halo effects. In addition, the color of the image is too saturated. Finally, the images restored using the method of Berman [9] show obvious color distortion. In addition, the sun in Figure 12d is enlarged. Besides, it should be noted that the method of He [6] performs better for testing most images, but when it comes to the images with a large sky area, the retored results will have serious color distortion. As opposed to the four methods, the color using our method is closer to that of the input image. For example, the lawn in the last image restored using our method is green. Not only does our method achieve complete dehazing, it effectively avoids halo effects and color distortion.

**Figure 13:**
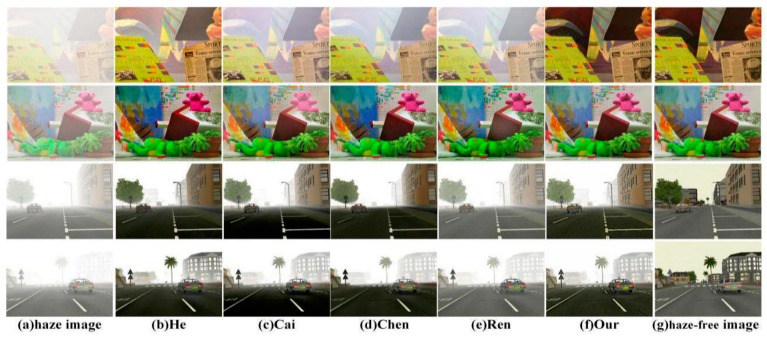
A comparison of synthetic images.

To more comprehensively evaluate our method, we compare different experimental results by testing synthetic haze images, which were extracted from the Road Image Databases [21] and stereo images [13]. Through the comparison in Figure 13, it is apparent that the images restored using the method of He [6] and our method are the best. The method of Cai [17] causes the color in the restored image to be supersaturated due to underextraction of some features, such as the darker road in Figure 13c. The images recovered using the methods of Chen [18] and the Ren [19] have more residual mist. In addition, the experimental results from the two methods show great differences from the real haze-free images in the Road Image Databases. In contrast, our method also achieved satisfactory results for synthetic images. On the one hand, the color of our experimental results is closer to the real haze-free images than when using the method of He [6]. On the other hand, there is no halo effect in our experimental results, which reflects the advantages of the method in detail recovery and better color preservation.

### 4.2. Quantitative Results

To further verify the effectiveness of our method, we used the information entropy [22,23] of the image to evaluate the performance of the method from an objective perspective. Information entropy represents the amount of information contained in the image. The larger the value, the more details the experimental results include. As shown in Table 1, the entropy of images restored using our method are higher than others. It shows that our method outperforms most of other methods in detail recovery. The effectiveness of our method is further verified by the quantitative analysis, which fully demonstrates the advantages of our method of detail recovery.

To further verify the effectiveness of our method for synthetic images, we analyze the experimental results in Figure 13 through the information entropy and the peak signal noise ratio (PSNR). PSNR reflects the integrity of image structure. As shown in the Table 2 and Table 3, our method and He [6] have advantages over other methods in information entropy. However, when it comes PSNR, He [6] for partial images has low value, which shows that the applicability of this method is poor. In contrast, our method obtains higher values for all results in PSNR, the restored images are clearer and natural, and the effectiveness of image dehazing is improved.

**Figure 14:**
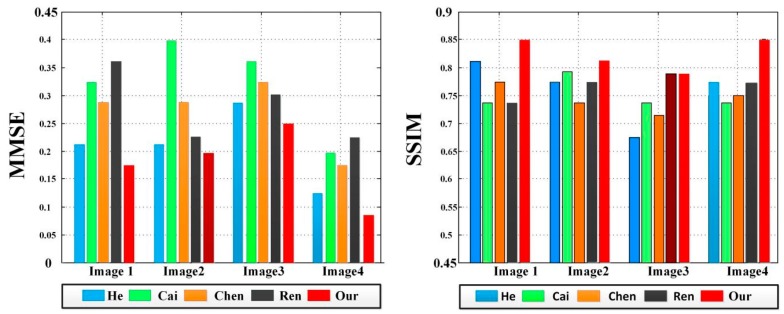
A comparison of MMSE and SSIM in Figure 13.

In addition, the minimum mean square error (MMSE) and structural similarity index (SSIM) are introduced to further illustrate the advantages and disadvantages of each method [24,25], which can help us comprehensively compare the difference among the restored images. As shown in Figure 14, the experimental results using the methods of Cai [17], Chen [18] and Ren [19] in Figure 13 have higher MMSE, indicating that they do not completely achieve dehazing. Meanwhile, although the experimental results using the method of He [6] have lower MMSE, the SSIM are also lower. In contrast, the MMSE of our method is low, indicating that the details of the restored image are clear. At the same time, the SSIM of our method is the highest among all results. In conclusion, the reliability of our method is further verified by quantitative analysis.

### 4.3. Computational Complexity

To compare and analyze the efficiency of each method, we select images of different sizes to test each method and record its running time. As we can see in Table 4, The running time of our method is longer than that of Ren [19] and is close to that of Cai [17]. However, the experimental results of our method are superior to those of Ren [19] and Cai [17] obviously. Since the Chen [18] uses 500 iterations to estimate the transmission, its computational complexity is higher than others. What’s more, He [6] has a high computational complexity when refining the transmission through soft matting. In comparison, although our method is not the fastest, it realizes the effective dehazing without sacrificing the running time.

## 5. Conclusions

In summary, our method has a significantly improves the image quality of sensors in foggy conditions. To obtained the haze-free image, we estimated the transmission according to the features in the image, and constructed a mapping relationship between the features and the transmission map using GAN, which effectively avoided color distortion and retained image details. The experimental results in this paper are better when compared with most of other methods. However, our method is inferior to the sensor image obtained in dense fog. Our future focus will be on the dehazing of images with dense fog.

## Figures and Tables

**Table 1:** The information entropy from images shown in Figure 12.

Haze Image	Zhu	Tang	Berman	He	Our
Image 1	8.432	8.541	7.582	8.725	9.451
Image 2	8.626	7.896	6.750	8.086	9.275
Image 3	8.241	8.452	8.527	9.103	9.263

**Table 2:** The information entropy from images shown in Figure 13.

Haze Image	He	Cai	Chen	Ren	Our
Image 1	7.2846	6.8753	6.7152	5.9875	7.4783
Image 2	7.1342	6.7883	7.2936	6.9983	7.4982
Image 3	7.4568	7.3512	7.6589	7.4537	7.9375
Image 4	7.6639	7.5697	6.2589	7.2358	7.8165

**Table 3:** The PSNR from images shown in Figure 13.

Haze Image	He	Cai	Chen	Ren	Our
Image 1	11.1765	14.2586	10.5896	10.1568	16.8974
Image 2	17.6538	15.3692	18.5693	14.2568	19.5683
Image 3	14.9577	17.6598	17.2563	16.8593	17.5836
Image 4	22.0103	15.6984	15.1750	19.9872	21.2258

**Table 4:** The execution time of different size of images.

Image Size	He	Cai	Chen	Ren	Our
440 × 320	9.563 s	3.124 s	50.432 s	1.947 s	5.016 s
670 × 480	11.768 s	4.598 s	106.398 s	3.685 s	6.697 s
1024 × 768	35.269 s	8.796 s	180.148 s	5.984 s	10.896 s
1430 × 1024	72.531 s	20.015 s	250.654 s	11.369	22.573 s
